# Sweroside Prevents Non-Alcoholic Steatohepatitis by Suppressing Activation of the NLRP3 Inflammasome

**DOI:** 10.3390/ijms21082790

**Published:** 2020-04-17

**Authors:** Gabsik Yang, Joo Hyeon Jang, Sung Wook Kim, Sin-Hee Han, Kyung-Ho Ma, Jae-Ki Jang, Han Chang Kang, Yong-Yeon Cho, Hye Suk Lee, Joo Young Lee

**Affiliations:** 1 BK21plus Team, College of Pharmacy, The Catholic University of Korea, Bucheon 14662, Korea; yangboncho@gmail.com (G.Y.); jangzoo@catholic.ac.kr (J.H.J.); sheep.sw91@gmail.com (S.W.K.); hckang@catholic.ac.kr (H.C.K.); yongyeon@catholic.ac.kr (Y.-Y.C.); sianalee@catholic.ac.kr (H.S.L.); 2 Department of Pharmacology, College of Korean Medicine, Woosuk University, Jeonbuk 55338, Korea; 3 Department of Herbal Crop Research, National Institute of Horticultural & Herbal Science, Rural Development Administration, Eumsung 27709, Korea; herbman@korea.kr (S.-H.H.); khma@korea.kr (K.-H.M.); changjk@korea.kr (J.-K.J.)

**Keywords:** inflammasome, steatohepatitis, liver, mitochondria, inflammation

## Abstract

Non-alcoholic steatohepatitis (NASH), a type of non-alcoholic fatty liver disease, is characterized as steatosis and inflammation in the liver. NLRP3 inflammasome activation is associated with NASH pathology. We hypothesized that suppressing the NLRP3 inflammasome could be effective in preventing NASH. We searched substances that could inhibit the activation of the NLRP3 inflammasome and identified sweroside as an NLRP3 inhibitor. We investigated whether sweroside can be applied to prevent the pathological symptoms associated with NASH in a methionine–choline-deficient (MCD) diet-induced NASH mouse model. The activation of the NLRP3 inflammasome was determined by detecting the production of caspase-1 and IL-1β from pro-caspase-1 and pro-IL-1β in primary mouse macrophages and mouse liver. In a NASH model, mice were fed an MCD diet for two weeks with daily intraperitoneal injections of sweroside. Sweroside effectively inhibited NLRP3 inflammasome activation in primary macrophages as shown by a decrease in IL-1β and caspase-1 production. In a MCD diet-induced NASH mouse model, intraperitoneal injection of sweroside significantly reduced serum aspartate transaminase and alanine transaminase levels, hepatic immune cell infiltration, hepatic triglyceride accumulation, and liver fibrosis. The improvement of NASH symptoms by sweroside was accompanied with its inhibitory effects on the hepatic NLRP3 inflammasome as hepatic IL-1β and caspase-1 were decreased. Furthermore, sweroside blocked de novo synthesis of mitochondrial DNA in the liver, contributing to suppression of the NLRP3 inflammasome. These results suggest that targeting the NLRP3 inflammasome with sweroside could be beneficially employed to improve NASH symptoms.

## 1. Introduction

Non-alcoholic fatty liver disease (NAFLD) is a condition with excess fat deposited in the liver without heavy alcohol drinking. NAFLD is one of the most prevalent liver diseases worldwide and affects more than a third of the western population [1]. NAFLD includes simple fatty liver with little inflammation or liver cell damage as well as non-alcoholic steatohepatitis (NASH), the severe form of NAFLD accompanying liver inflammation and damage associated with fat accumulation in the liver. The pathological symptoms of NASH progress to fibrosis, which in turn is exacerbated to cirrhosis or hepatocellular carcinoma [2]. Therefore, a therapeutic strategy to regulate NAFLD, especially the NASH condition, would be beneficial for preventing severe liver diseases and consequent the mortality rate. 

NAFLD is well known to be associated with metabolic diseases such as obesity, type 2 diabetes, hyperlipidemia, hypertension, and insulin resistance [2,3]. Despite these associations, the specific mechanisms governing NAFLD-related metabolic diseases and liver pathology remain unclear. Recent evidence indicates the important role of the NLRP3 inflammasome in the pathological development and progression of NAFLD and NASH. The NLRP3 inflammasome is a multiprotein complex which is composed of NLRP3, ASC, and pro-caspase-1, to activate caspase-1 to produce mature IL-1β. In addition to pathogen invasion, endogenous damage-associated signals induce the activation of the NLRP3 inflammasome [4,5]. The activation of the NLRP3 inflammasome and the consequent secretion of IL-1β are well correlated with a number of chronic diseases including NAFLD and NASH. The hepatic expression of the NLRP3 inflammasome components is increased in liver diseases [6,7]. IL-1β is one of the first signaling cytokines associated with NASH [8,9]. NASH induced by a methionine–choline-deficient (MCD) diet is associated with caspase-1 activation in the liver [10]. NLRP3 knock-out mice are protected against NASH-induced steatosis, liver injury, immune cell infiltration, and inflammation in the choline-deficient amino acid-defined diet-induced NASH state [11]. Experimental and clinical evidence shows that the expression of NLRP3 in hepatocytes increases during the development of NAFLD [8]. Moreover, mice deficient in NLRP3 or its essential components, ASC or caspase-1, are protected from fatty liver disease caused by the atherogenic and MCD diet [8]. These findings indicate that the activation of the NLRP3 inflammasome is involved in triggering liver inflammation in NASH. Therefore, we seek to identify substances that could inhibit the activation of the NLRP3 inflammasome and investigate whether the NLRP3 inhibitors can be applied to prevent the pathological symptoms associated with NASH. 

Sweroside is known to possess a wide variety of biological activities, including wound healing, as well as anti-bacterial, anti-fungal, and anti-allergic effects, and is typically considered in the treatment of hepatobiliary disorders [12]. Pre-clinical experiments in rats have shown that the administration of sweroside effectively protects against chemical-induced liver fibrosis and injury [13,14]. However, it has not been studied whether sweroside affects activation of the NLRP3 inflammasome and is effective to prevent NASH. In this study, we investigated whether sweroside inhibited activation of the NLRP3 inflammasome, leading to the alleviation of the pathological symptoms of NASH in primary macrophages and an animal NASH model. 

## 2. Results

### 2.1. Sweroside Inhibits the Activation of the NLRP3 Inflammasome in Primary Macrophages

**Figure 1:**
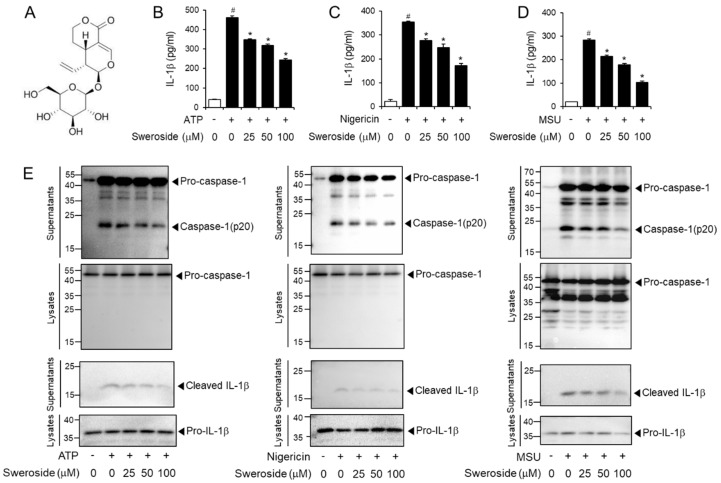
Sweroside inhibits the activation of the NLRP3 inflammasome in primary macrophages. (**A**) Chemical structure of sweroside. (**B**–**D**) Bone marrow-derived macrophages (BMDMs) were primed with LPS (100 ng/mL) for 4 h. The cells were treated with sweroside for 1 h and then stimulated with (**B**) ATP (5 mM) for 2 h, (**C**) nigericin (10 µM) for 16 h, or (**D**) MSU (500 µg/mL) for 6 h. Cell culture supernatants were analyzed for secreted IL-1β by ELISA. The values represent the means ± SEM (*n* = 3). #, significantly different from vehicle alone, *p* < 0.05. *, significantly different from ATP, nigericin, or MSU alone, *p* < 0.05. (**E**) BMDMs were primed with LPS (100 ng/mL) for 4 h. The cells were treated with sweroside for 1 h and then stimulated with ATP (5 mM) for 1 h, nigericin (10 µM) for 1 h, or MSU (500 µg/mL) for 4.5 h. The cell culture supernatants and cell lysates were immunoblotted for pro-caspase-1, caspase-1(p20), pro-IL-1 β, and IL-1 β.

To investigate whether sweroside (Figure 1A) inhibited the activation of the NLRP3 inflammasome, the extracellular secretion of mature IL-1β was determined in mouse bone marrow-derived primary macrophages (BMDMs) as a hallmark of NLRP3 inflammasome activation. Sweroside decreased IL-1β secretion from BMDMs stimulated with ATP, a representative agonist of the NLRP3 inflammasome as determined by enzyme-linked immunosorbent assay (ELISA) (Figure 1B). Similarly, sweroside reduced IL-1β secretion induced by other NLRP3 agonists, nigericin and monosodium uric acid (MSU) crystals in BMDMs (Figure 1C,D). Cleavage of pro-caspase-1 to caspase-1 (p20) is another indicator of NLRP3 inflammasome activation. Sweroside blocked the degradation of pro-caspase-1 to caspase-1 (p20) induced by ATP in BMDMs as shown by immunoblotting (Figure 1E). Consistently, sweroside suppressed nigericin- or MSU-induced degradation of pro-caspase-1 to caspase-1 (p20) in BMDMs (Figure 1E). These results show that sweroside suppresses the activation of the NLRP3 inflammasome induced by various agonists in primary macrophages. We examined the impact of sweroside on cell death in ATP- or nigericin-treated BMDMs using MTT assay. Sweroside treatment did not induce significant cytotoxicity up to 100 µM in ATP- or nigericin-treated BMDMs (Figure S1A,B). In addition, no significant cell death was observed by sweroside treatment alone in BMDMs (Figure S1C).

To address the specificity of sweroside’s inhibitory effect, we examined the effects of sweroside on other inflammasome activations such as AIM2 and NLRC4. The results show that sweroside did not block poly dA:dT-induced production of caspase-1 and IL-1β in macrophages (Figure S2A). Similarly, sweroside did not suppress flagellin-induced production of caspase-1 and IL-1β in macrophages (Figure S2B). These results show that sweroside does not inhibit the activation of AIM2 and NLRC4 in macrophages.

### 2.2. Sweroside Blocks the Formation of ASC Specks in Primary Macrophages

**Figure 2:**
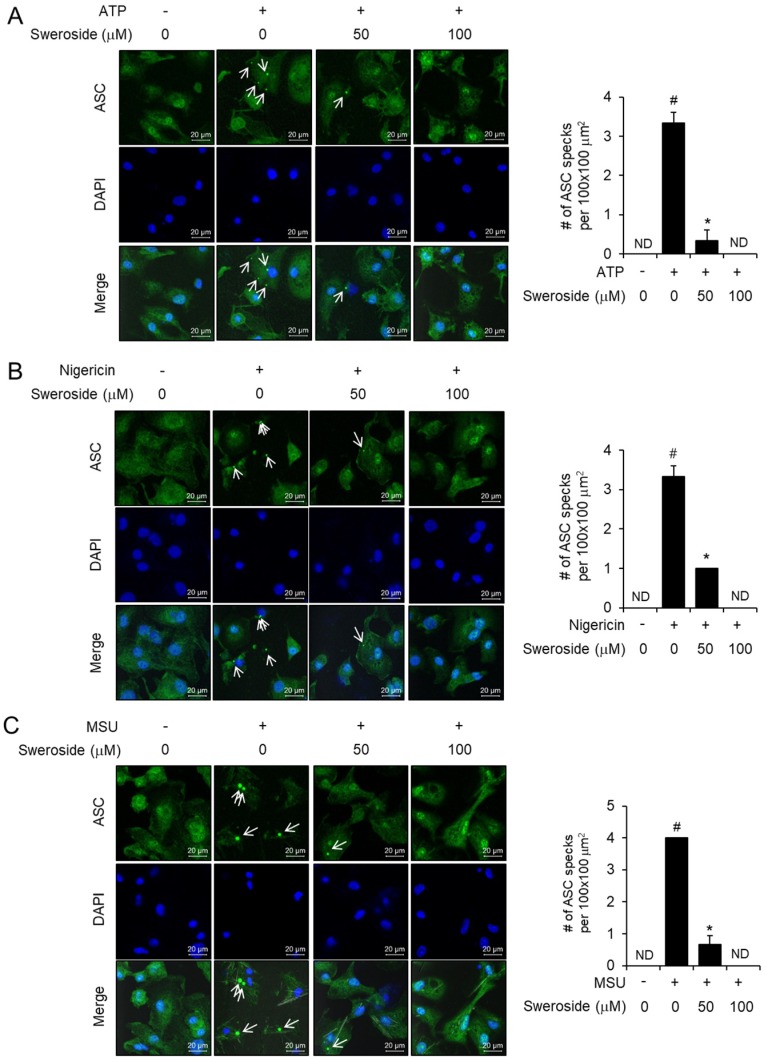
Sweroside blocks the formation of ASC specks in primary macrophages. (**A**–**C**) Bone marrow-derived macrophages (BMDMs) were fixed, permeabilized, and stained for ASC (green). The nuclei were stained with 4′,6-diamidino-2-phenylindole (DAPI: blue). The arrows indicate ASC specks. The number of ASC specks per 100 × 100 µm^2^ was obtained from different fields of view and is presented as a bar graph. The values represent the means ± SEM (*n* = 3). #, significantly different from vehicle alone, *p* < 0.05. *, significantly different from ATP, nigericin, or MSU alone, *p* < 0.05. ND, not detected. Scale bars = 20 µm.

ASC is an adaptor composing the NLRP3 inflammasome complex. Upon agonist stimulation, NLRP3 combines with ASC, inducing the formation of ASC specks, which recruit pro-caspase-1 for auto-activation of caspase-1. Therefore, ASC speck formation is a prerequisite for pro-caspase-1 degradation and auto-activation. Confocal microscopy analysis show that ATP induced the speck formation of ASC in BMDMs, while sweroside reduced ATP-induced formation of ASC specks (Figure 2A). Furthermore, sweroside blocked the formation of ASC specks induced by nigericin or MSU crystals (Figure 2B,C). These results confirm the inhibitory effects of sweroside on the NLRP3 inflammasome. The results suggest that sweroside affects the upstream step of ASC speck formation. 

### 2.3. Sweroside Alleviates Hepatic Inflammation and Fat Accumulation in Mice Fed a Methionine–Choline-Deficient Diet

**Figure 3:**
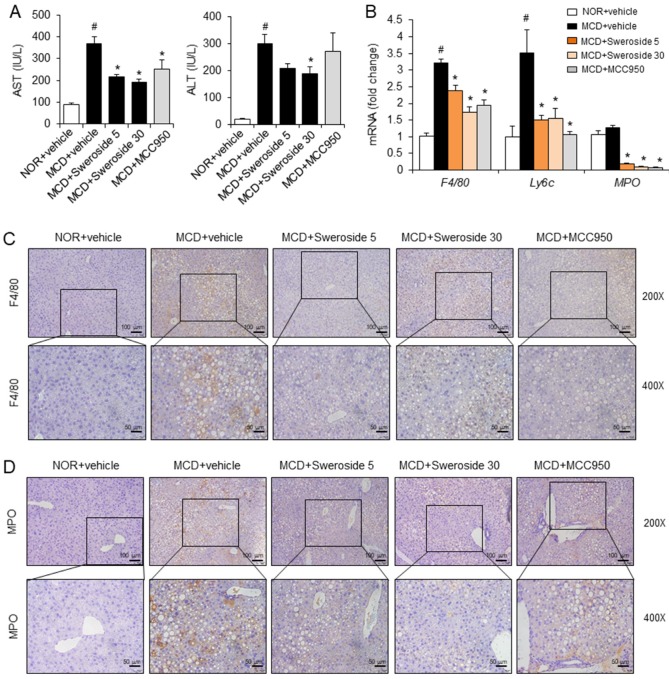
Sweroside alleviates hepatic inflammation in mice fed an MCD diet. C57BL/6 mice were fed a normal diet (NOR) or methionine-choline-deficient diet (MCD) for two weeks. Sweroside (5, 30 mg/kg) or MCC950 (20 mg/kg) was administered with daily intraperitoneal injections with the diets. (**A**) Serum AST and ALT levels were determined. (**B**) Hepatic mRNA levels for total macrophages (*F4/80*), inflammatory macrophages (*Ly6c*), and neutrophils (*MPO*). (**C**,**D**) Representative immunohistochemistry liver sections stained for F4/80 and MPO (200X and 400X). For A and B, the values represent means ± SEM (*n* = 8). #, significantly different from NOR + vehicle, *p* < 0.05. *, significantly different from MCD + vehicle, *p* < 0.05.

The activation of the NLRP3 inflammasome plays a critical role in triggering liver inflammation, which is an important feature of NASH [11]. Therefore, we investigated whether inhibition of the NLRP3 inflammasome by sweroside could lead to the prevention of liver inflammation in a NASH state. We employed a MCD diet model, which is a widely used dietary model to induce NASH status [15]. Plasma levels of aspartate aminotransferase (AST) and alanine aminotransferase (ALT), which are liver inflammation indicators, significantly increased when mice were on the MCD diet for two weeks. Intraperitoneal injection of sweroside, 5 and 30 mg/kg, to the MCD diet-fed mice notably reduced both AST and ALT levels (Figure 3A). MCC950, an NLRP3 inflammasome inhibitor, was used as a positive control. Intraperitoneal injection of MCC950 (20 mg/kg) reduced AST levels induced by the MCD diet while it did not decrease ALT levels (Figure 3A). Infiltration of total macrophages, inflammatory macrophages, and neutrophils in the liver was determined by measuring hepatic mRNA levels of F4/80, Ly6c, and MPO, respectively. Infiltration of total macrophages (F4/80) and inflammatory macrophages (Ly6c) in the liver significantly increased in MCD diet-fed mice as compared with normal chow diet (NOR)-fed mice while infiltration of neutrophils (MPO) increased very slightly (Figure 3B). Interestingly, infiltration of total macrophages (F4/80), inflammatory macrophages (Ly6c), and neutrophils (MPO) was downregulated by 5 and 30 mg/kg of sweroside treatment (Figure 3B). Similarly, MCC950 treatment reduced the hepatic mRNA levels of F4/80, Ly6c, and MPO increased by the MCD diet (Figure 3B). Immunohistochemical analysis showed that hepatic infiltration of total macrophages (F4/80) and neutrophils (MPO) was induced by the MCD diet (Figure 3C,D). In contrast, sweroside treatment reduced the immunohistochemical staining for F4/80 and MPO, showing a decrease of macrophage and neutrophil infiltration (Figure 3C,D). Similarly, MCC950 decreased the infiltration of macrophages and neutrophils induced by the MCD diet (Figure 3C,D). These results show that sweroside treatment reduced hepatic inflammation induced by an MCD diet in mice. 

**Figure 4:**
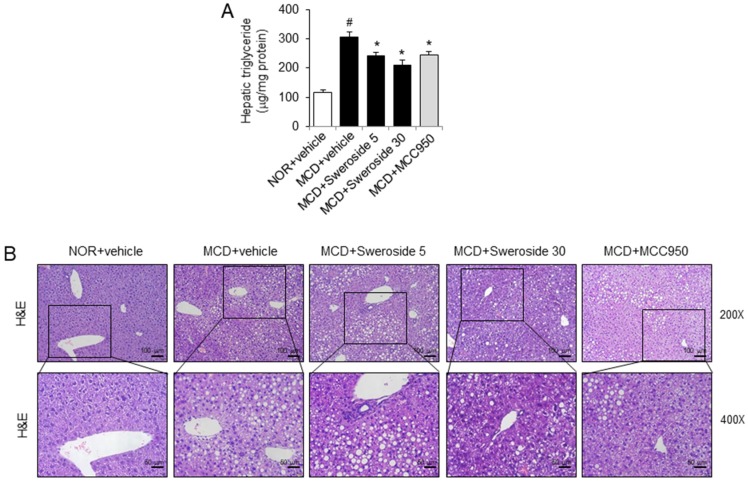
Sweroside reduces hepatic triglyceride accumulation in mice fed an MCD diet. Samples were obtained from the mice described in Figure 3. (**A**) Hepatic triglyceride levels. The values represent means ± SEM (*n* = 8). #, significantly different from NOR + vehicle, *p* < 0.05. *, significantly different from MCD + vehicle, *p* < 0.05. (**B**) Representative H&E-stained sections of liver tissue (200× and 400×). The hepatic steatosis and infiltrated neutrophils were identified via H&E staining. The purple dot indicates neutrophils.

As hepatic steatosis is another hallmark of NASH, we determined if sweroside treatment could reduce hepatic fat accumulation in MCD diet-fed mice. Triglyceride accumulation in the liver was significantly increased in mice fed the MCD diet (Figure 4A). Intraperitoneal injection of sweroside, 5 and 30 mg/kg, markedly reduced hepatic triglyceride accumulation induced by the MCD diet (Figure 4A). Similarly, MCC950 decreased triglyceride accumulation in the liver induced by the MCD diet (Figure 4A). Hematoxylin and eosin staining confirmed that hepatic steatosis was induced by the MCD diet as lipid droplets in the liver were increased (Figure 4B). Sweroside and MCC950 marginally reduced the amount of hepatic lipid droplets (Figure 4B).

### 2.4. Sweroside Reduces Liver Fibrosis in Mice Fed a MCD Diet

**Figure 5:**
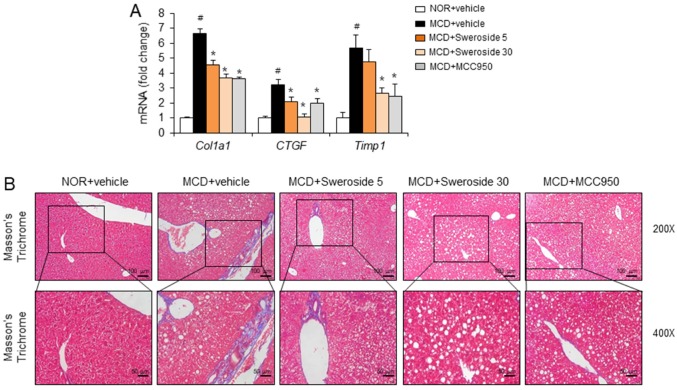
Sweroside alleviates hepatic fibrosis in mice fed an MCD diet. Samples were obtained from the mice described in Figure 3. (**A**) Hepatic mRNA expression of collagen type 1 (*Col1a1*), connective tissue growth factor (*CTGF*), and a tissue inhibitor of matrix metalloproteinase 1 (*Timp1*). The values represent means ± SEM (*n* = 8). #, significantly different from NOR + vehicle, *p* < 0.05. *, significantly different from MCD + vehicle, *p* < 0.05. (**B**) Representative pictures of Masson’s trichrome-stained liver sections (200× and 400×).

Liver fibrosis with collagen deposition occurs as the NASH state progresses. Therefore, we further examined whether sweroside could improve liver fibrosis. The mRNA levels of collagen type 1 (*Col1a1*), connective tissue growth factor (*CTGF*), and tissue inhibitor of matrix metalloproteinase 1 (*Timp1*), which are indicators of liver fibrosis, were measured. Compared to livers from normal chow diet-fed mice, mRNA levels of liver fibrosis markers were significantly upregulated in MCD diet-fed mice (Figure 5A). In contrast, sweroside treatment greatly reduced the expression of liver fibrosis markers increased by the MCD diet (Figure 5A). Masson’s trichrome staining, which shows collagen deposition, was significantly enhanced in livers from MCD diet-fed mice (Figure 5B). In contrast, sweroside treatment decreased the degree of Masson’s trichrome staining, showing that sweroside decreased collagen deposition in the liver induced by an MCD diet (Figure 5B). Similarly, MCC950 reduced mRNA levels of liver fibrosis markers and the amount of Masson’s trichrome staining increased by the MCD diet (Figure 5A,B). The results show that sweroside is effective in blocking hepatic fibrosis in mice fed an MCD diet.

### 2.5. Sweroside Suppresses the Activation of Hepatic NLRP3 Inflammasome in Mice Fed an MCD Diet

**Figure 6:**
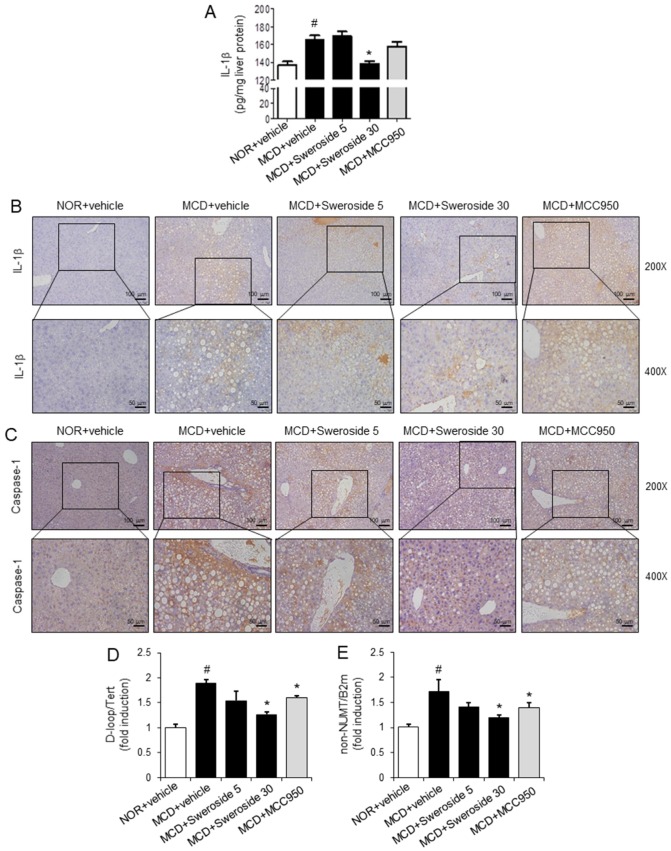
Sweroside suppresses the activation of the hepatic NLRP3 inflammasome in mice fed an MCD diet. Samples were obtained from the mice described in Figure 3. (**A**) IL-1β protein levels in liver homogenates were measured by ELISA. The values represent means ± SEM (*n* = 8). (**B**,**C**) Representative immunohistochemistry staining for IL-1 β and caspase-1 in the liver (200× and 400×). (**D**,**E**) Relative total mtDNA amounts in liver tissues were quantified by quantitative PCR with primers specific for the mitochondrial D-loop region (D-loop) or a region of mtDNA that is not inserted in nuclear DNA (non-NUMT). The amount of nuclear DNAs (Tert, B2m) was measured to normalize mtDNA production. The values represent means ± SEM (*n* = 4). For A, D, and E, #, significantly different from NOR + vehicle, *p* < 0.05. *, significantly different from MCD + vehicle, *p* < 0.05.

We next investigated whether the protective effects of sweroside on NASH symptoms induced by an MCD diet in mice were mediated by its inhibitory effects on the NLRP3 inflammasome in the liver. Hepatic levels of IL-1β, which is a hallmark of NLRP3 inflammasome activation, were increased in MCD-diet-fed mice (Figure 6A). Intraperitoneal injection of sweroside, 30 mg/kg, significantly reduced hepatic IL-1β levels increased by the MCD diet to the normal level (Figure 6A). MCC950 20 mg/kg was only marginally effective to reduce IL-1β levels (Figure 6A). In addition, immunohistochemical analysis show that the MCD diet increased the hepatic expression of IL-1β and caspase-1, while sweroside treatment decreased MCD diet-induced expression of IL-1β and caspase-1 in the liver (Figure 6B,C). These results show that sweroside treatment suppressed the activation of the NLRP3 inflammasome in livers of MCD diet-fed mice. The results suggest that the suppressive effects of sweroside on NASH symptoms induced by the MCD diet are linked to the inhibition of the hepatic NLRP3 inflammasome. 

Oxidized mitochondrial DNA (mtDNA) induces activation of the NLRP3 inflammasome [16]. Blockade of mtDNA synthesis reduced IL-1β production induced by NLRP3 activators such as ATP, nigericin, and MSU [17]. Therefore, we investigated whether sweroside blocked de novo synthesis of mtDNA in the liver. The amounts of mtDNA such as the mitochondrial D-loop region (D-loop) or a region of mtDNA that is not inserted in nuclear DNA (non-NUMT) were determined and normalized by the amount of nuclear DNAs (Tert, B2m). The MCD diet increased the amount of mtDNA (D-loop, non-NUMT) in liver tissues, while sweroside treatment resulted in the reduction of mtDNA production induced by the MCD in liver tissues (Figure 6D,E). These findings suggest that suppression of the NLRP3 inflammasome by sweroside is mediated by the reduction of mitochondrial DNA synthesis. 

## 3. Discussion

Our results demonstrate that inhibition of the NLRP3 inflammasome could provide a potential therapeutic tactic to treat or prevent the pathological symptoms of NASH. The NLRP3 inflammasome plays an important role in the progression of NASH [11]. IL-1β, the major product of NLRP3 inflammasome activation, is a potent inflammatory cytokine, inducing the death of hepatocytes, inflammation, and activation of stellate cells [18]. IL-1β induces accumulation of lipids and inflammation in liver tissues [19]. Caspase-1, which is activated upon NLPR3 inflammasome activation, regulates fibrogenesis [10]. Our results show that sweroside blocks the activation of caspase-1 and the production of IL-1β, in an in vitro cell system and an in vivo experimental NASH model. The inhibitory effects of sweroside on the NLRP3 inflammasome in macrophages and liver tissues are linked to its preventive effects on the pathological symptoms of NASH, such as hepatic inflammation, triglyceride accumulation, and fibrosis. 

Both nonalcoholic fatty liver disease and nonalcoholic steatohepatitis are caused by factors other than alcohol. Therefore, an effective treatment may target key events such as molecular and cellular processes of inflammatory responses. The preventive strategy for the public represents the control of a healthy lifestyle with diet and exercise. The pharmacological approach includes the regulation of peroxisome proliferator–activator receptors (PPAR), a farnesoid X receptor (FXR)-bile acid axis, lipid remodeling, incretin system, inflammation, apoptosis, and oxidative stress [20,21]. Recently, gut- and microbiome-related treatment and an anti-fibrosis treatment have drawn much attention. In this study, we present sweroside as an effective pharmacological approach. 

An MCD diet is a widely used dietary model that induces NASH status with hepatic steatosis, inflammation, and fibrosis [15]. Liver damage mediated by inflammation is a main feature caused by the MCD diet [22,23]. In addition, an MCD diet results in severe fibrosis, derived from collagen deposition in the liver [24,25]. 

The underlying mechanism for the development and progression of NAFLD is complex and multifactorial. Different theories have been formulated, leading initially to the ‘two hits hypothesis’. According to this, hepatic accumulation of lipids secondary to a sedentary lifestyle, high fat diet, obesity, and insulin resistance, acts as the first hit, sensitizing the liver to further insults acting as a ‘second hit’. The ‘second hit’ activates inflammatory cascades and fibrogenesis [26]. The pro-inflammatory cytokines and adipokines have been implicated in the pathogenesis of NAFLD. In addition, cytokines (TNF-α, IL-6, and IL-1β) may play a role in the hepatic and systemic insulin resistance associated with NASH [27]. We imposed the MCD diet for two weeks to determine the effects of IL-1β through NLRP3 regulation, before severe fibrosis progressed. In vivo treatment of sweroside is effective to reduce the main characteristics of NASH such as hepatic steatosis, inflammation, and fibrosis induced by an MCD diet. The results suggest the possibility of an effective clinical application of sweroside to NASH patients. Consistently, Yang Q et al. reported that sweroside reduced hepatic lipid accumulation and serum lipid levels with decreased hepatic and serum levels of pro-inflammatory cytokines and chemokines in high fat diet-induced obese mice, showing that sweroside improved NAFLD symptoms mediated through the regulation of lipid metabolism- and inflammation-related genes [28].

Activation of the NLRP3 inflammasome involves two signals. The first signal induces pro-IL-1β expression by a TLR agonist. The second signal is triggered by the NLRP3 inflammasome agonists to form the inflammasome complex resulting in the activation of caspase-1 to cleave pro-IL-1β to the mature form of IL-1β [29]. Wang et al. reported that sweroside suppressed LPS-induced expression of pro-inflammatory cytokines including IL-1β, TNF-α, and IL-6 mediated through the downregulation of NF- κB activation in a murine macrophage cell line (RAW264.7) [30]. The suppressive effect of sweroside on NF-κB activation is also shown in IL-1β-stimulated rat articular chondrocytes, culminating in the decreased expression of iNOS and COX-2 [31]. Since the inhibitory effect of sweroside on LPS signaling has been studied, we intended to focus on the regulation of the NLRP3 inflammasome activity by sweroside rather than the LPS priming signal. Our results demonstrate that anti-inflammatory activity of sweroside is mediated through the blockade of the NLRP3 inflammasome complex formation resulting in the reduction of IL-1β maturation and secretion. It is possible that in vivo anti-inflammatory activity of sweroside may be attributed to the net outcome of its suppressive effects on both the LPS priming step and the NLRP3 inflammasome, potentiating the anti-inflammatory efficacy of sweroside. 

## 4. Materials and Methods

### 4.1. Ethics Statement

All animals received humane care according to the criteria outlined in the “Guide for the Care and Use of Laboratory Animals” prepared by the National Academy of Sciences and the National Institutes of Health (NIH publication 86–23 revised 1985). All experimental procedures were carried out in accordance with the protocols approved by the Institutional Animal Care and Use Committee (IACUC) of the Catholic University of Korea (permission# 2016-004-02, approval date 16 July 2018).

### 4.2. Animals and Cell Culture

C57BL/6 mice (male) were obtained from Orient bio (Seoul, Korea) and were acclimated in specific pathogen-free conditions in an animal facility for at least one week before experimentation. The mice were housed in a 12:12-h light/dark cycle, temperature (23 ± 3 °C) and humidity (40–60%) controlled room. All mice were allowed ad libitum access to their diet and water during the experimental period. Mice were acclimated in the animal facility for at least one week before the experiments. Mice of individual experimental groups in each experiment were of similar age and weight and were randomly allocated to treatment groups. Investigators were blinded to the treatment throughout the experiment. 

Bone marrow-derived primary macrophages (BMDMs) were prepared after bone marrow was isolated from C57BL/6 mice as described previously [32]. Macrophages were cultured in Dulbecco’s modified Eagle medium containing 10% (*v/v*) fetal bovine serum (Invitrogen, Carlsbad, CA, USA), 10,000 units/mL penicillin, and 10,000 μg/mL streptomycin. 

### 4.3. Reagents

Purified lipopolysaccharides (LPS) from *Escherichia coli* were obtained from List Biological Laboratory (Campbell, CA, USA) and dissolved in endotoxin-free water. Sweroside (purity: ≥98%) was purchased from Chemfaces (Wuhan, China). ATP, monosodium urate (MSU), and MCC950 were purchased from Invivogen (Carlsbad, CA, USA). Nigericin was obtained from Sigma–Aldrich (St Louis, MO, USA). Antibodies for mouse caspase-1 or ASC were obtained from Santa Cruz Biotechnology (Santa Cruz, CA, USA). Antibody for NLRP3 was purchased from Adipogen (San Diego, CA, USA). Antibody for IL-1β was obtained from R&D Systems (Minneapolis, MN, USA). 

### 4.4. Analysis of Inflammasome Activation

This was performed as described previously [33]. BMDMs were seeded at 2 × 10^6^ cells/well in 6-well plates for immunoblot assay and 4 × 10^5^ cells/well in 96-well plates for ELISA. BMDMs were primed with LPS for 4 h. To exclude the effect of sweroside on the LPS priming step, sweroside was added after washing out the LPS with phosphate-buffered saline. After the cells were treated with sweroside, cells were further stimulated with NLRP3 inflammasome activators such as ATP, nigericin, and MSU crystals in a serum-free medium. The cells were lysed in a RIPA buffer (50 mM Tris-HCl, pH 7.4, 1% NP-40, 0.25% sodium deoxycholate, 150 mM NaCl, 1 mM EGTA, 1 mM PMSF, 1 mM Na_3_VO_4_, 10 μg/mL aprotinin, 10 μg/mL leupeptin), and the supernatants were precipitated with methanol:chloroform (1:0.25), followed by centrifugation at 20,000× *g* for 10 min. The upper phase was discarded, and one volume of methanol was added. The mixture was centrifuged at 20,000× *g* for 10 min to obtain a protein pellet that was dried at room temperature and then resuspended in Laemmli buffer (0.25 M Tris-HCl, pH 6.8, 0.4% glycerol, 10% SDS, 0.2% 2-mercaptoethanol, 0.64% bromophenol blue). The samples were resolved with SDS-PAGE and subjected to immunoblotting assay [34].

### 4.5. Enzyme-Linked Immunosorbent Assay

The IL-1β levels in culture media and liver homogenate supernatants were determined using a DuoSet enzyme-linked immunosorbent assay (ELISA) kit (R&D systems, Minneapolis, MN, USA) according to the manufacturer’s instructions [35]. 

### 4.6. Confocal Microscopy Analysis

This was performed as described previously [36]. BMDMs were plated overnight on cover slides in 12-well plates. After the cells were fixed with methanol for 30 min, cells were blocked with 1% bovine serum albumin for 30 min and incubated with an anti-ASC antibody (Santa Cruz Biotechnology Inc., Santa Cruz, CA, USA) at 4 °C overnight. Cells were further incubated with an anti-rabbit IgG-FITC antibody (Invitrogen, Carlsbad, CA, USA) for 2 h at room temperature. Cells were co-stained with 4′,6-diamidino-2-phenylindole (DAPI, 1 μg/mL; Invitrogen, Carlsbad, CA, USA) for nuclei staining. Slides were mounted in fluorescent mounting medium (Vector laboratories, Burlingame, CA, USA), and samples were examined with an LSM710 laser scanning confocal microscope (Carl Zeiss, Oberkochen, Germany). Images were obtained and analyzed with ZEN2011 software (Carl Zeiss, Oberkochen, Germany). Three fields per group were counted and presented as the mean ± SEM. 

### 4.7. A Methionine–Choline-Deficient Diet Model

C57BL/6 mice (male, 11 weeks old) were randomly divided into five weight-matched groups (*n* = 8/group): normal chow diet (NOR), methionine-choline-deficient diet (MCD), MCD + sweroside 5 mg/kg, MCD + sweroside 30 mg/kg, and MCD + MCC950 20 mg/kg. Mice were fed an MCD for two weeks accompanied by a daily intraperitoneal injection of sweroside (5, 30 mg/kg) or MCC950 (20 mg/kg). At the end of the two-week period, all animals were fasted for 12 h. On the following day, mice were anesthetized with Zoletil (Virbac, Carros Cedex, France) and blood samples were collected by cardiac puncture. Liver tissues were excised, rinsed, weighed, and stored at −80 °C pending further analysis [37]. Serum levels of aspartate aminotransferase (AST) and alanine aminotransferase (ALT) were measured using an Express Plus Biochemistry analyzer (Chiron Diagnostics, Emeryville, CA). Triglyceride (TG) concentrations were measured by enzymatic assays (Sigma-Aldrich, St Louis, MO, USA).

### 4.8. Reverse Transcription and Quantitative Real-Time Polymerase Chain Reaction (qRT-PCR) Analysis 

Total RNAs from mouse livers or cultured cells were extracted using Trizol reagent (Invitrogen, Carlsbad, CA, USA) according to the manufacturer’s instructions. Reverse transcription and qRT-PCR analysis were performed as described previously [38]. The primers used were as follows: F4/80, 5′-TGA CTC ACC TTG TGG TCC TAA-3′ and 5′-CTT CCC AGA ATC CAG TCT TTC C-3′; Ly6c, 5′-GCA GTG CTA CGA GTG CTA TGG-3′ and 5′-ACT GAC GGG TCT TTA GTT TCC TT-3′; Mpo, 5′-AGT TGT GCT GAG CTG TAT GGA-3′ and 5′-CGG CTG CTT GAA GTA AAA CAG G-3′; Col1a1, 5′-GCT CCT CTT AGG GGC CAC T-3′ and 5′-CCA CGT CTC ACC ATT GGG G-3′; Ctgf, 5′-GGG CCT CTT CTG CGA TTT C-3′ and 5′-ATC CAG GCA AGT GCA TTG GTA-3′; Timp1, 5′-CTT GGT TCC CTG GCG TAC TC-3′ and 5′-ACC TGA TCC GTC CAC AAA CAG-3′; Actin, 5′-TCATGAAGTGTGACGTTGACATCCGT-3′ and 5′-TTGCGGTGCACGATGGAGGGGCCGGA-3′. 

Mitochondrial DNAs (mtDNAs) were quantified by quantitative PCR using primers specific for the mitochondrial D-loop region (D-loop) or a specific region of mtDNA that is not inserted in nuclear DNA (non-NUMT). Nuclear DNA encoding Tert or B2m was used for normalization. Primer sequences are as follows: D-loop, 5′-AATCTACCATCCTCCGTGAAACC-3′ and 5′-TCAGTTTAGCTACCCCCAAGTTTAA-3′; Tert, 5′-CTAGCTCATGTGTCAAGACCCTCTT-3′ and 5′-GCCAGCACGTTTCTCTCGTT-3′; B2m, 5′-ATGGGAAGCCGAACATACTG-3′ and 5′-CAGTCTCAGTGGGGGTGAAT-3′; non-NUMT, 5′-CTAGAAACCCCGAAACCAAA-3′ and 5′-CCAGCTATCACCAAGCTCGT-3′. The specificity of the amplified PCR products was assessed by melting curve analysis, and the gene expression was normalized to the corresponding actin levels. 

### 4.9. Histological Analysis

Liver tissue specimens were fixed in 10% buffered formalin, embedded in paraffin, cut into 3-μm-thick slices, and stained with hematoxylin and eosin (H&E) for the histological examination of liver tissue [39]. For detecting tissue protein expression, immunohistochemistry was performed [40]. To demonstrate fibrosis, liver sections were stained 2 h with Masson’s trichrome reagent [41].

### 4.10. Statistical Analysis

Data are expressed as the mean ± SEM. Comparisons of data between groups were performed by one-way ANOVA followed by Tukey’s multiple range test. Values of *p* < 0.05 were considered significant. Representative data are presented from two or three independent experiments.

## 5. Conclusions

In conclusion, this study demonstrates that targeting the NLRP3 inflammasome is beneficial to improve NASH symptoms such as inflammation, lipid accumulation, and fibrosis. Blockade of the NLRP3 inflammasome activation in macrophages and liver tissues by sweroside is well correlated with the improvement of hepatic inflammation, fat accumulation, and fibrosis induced by the MCD-diet. This study provides a novel therapeutic possibility to regulate the NASH symptoms with treatment of sweroside, mediated through the suppression of NLRP3 inflammasome activation.

## Supplementary Materials

Supplementary materials can be found at https://www.mdpi.com/1422-0067/21/8/2790/s1.

## Abbreviations

ALT	alanine aminotransferase
ASC	apoptosis-associated speck-like protein containing a CARD
AST	aspartate aminotransferase
BMDMs	bone marrow-derived primary macrophages
LPS	lipopolysaccharides
NAFLD	non-alcoholic fatty liver disease
NASH	non-alcoholic steatohepatitis
NLRP3	NOD-, LRR- and pyrin domain-containing protein 3
NOR	normal chow diet
MCD	methionine choline deficient
MSU	monosodium urate
mtDNAs	mitochondrial DNAs
TG	triglyceride
