# Supplementary material for: Sweroside Prevents Non-Alcoholic Steatohepatitis by Suppressing Activation of the NLRP3 Inflammasome
Source: Int J Mol Sci. 2020 Apr 17;21(8):2790. doi: 10.3390/ijms21082790 (PMC7216241; doi:10.3390/ijms21082790)
Supplement: Supplementary file 1 [file ijms-21-02790-s001.pdf]

International Journal of
*Molecular Sciences*

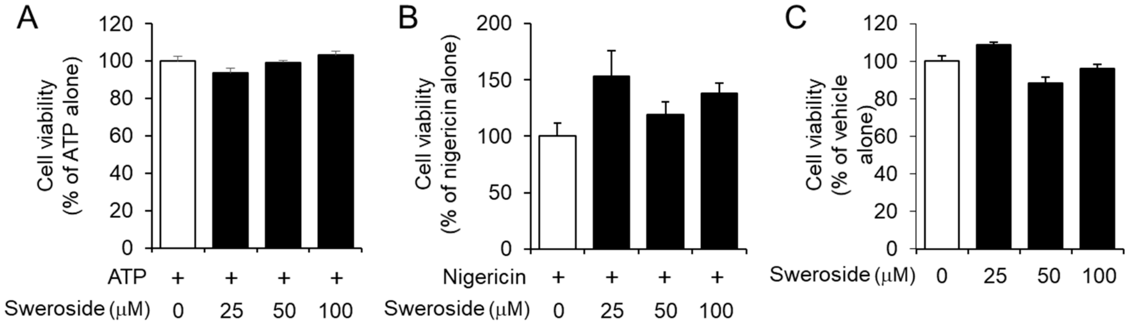


**Figure S1.** The effects of sweroside on cell viability. (**A–C**) Bone marrow-derived macrophages were treated with sweroside for 1 hour and further treated with (**A**) ATP (5 mM) for 2 hours or (**B**) nigericin (10 μM) for 16 hours. For **C**, sweroside was treated for 17 hours. Cell viability was determined by the MTT assay. Values indicate means ± SEM (*n* = 3).

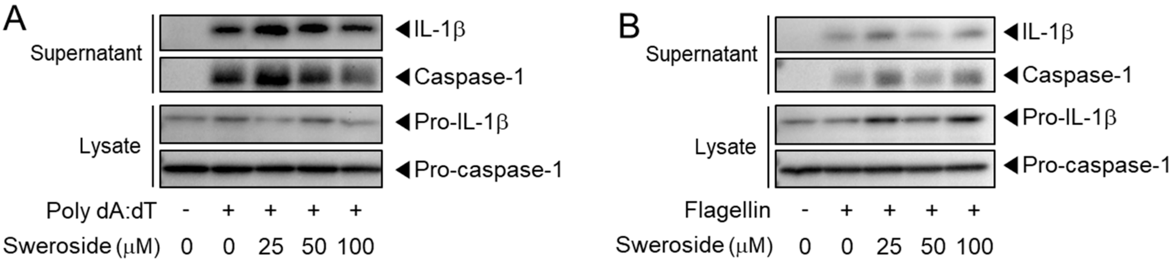


**Figure S2.** Effects of sweroside on the activation of AIM2 and NLRC4 inflammasomes in primary macrophages. (**A**,**B**) Bone marrow-derived macrophages were primed with LPS (500 ng/ml) for 4 hours. The cells were treated with sweroside for 1 hour and then stimulated with (**A**) poly dA:dT (1 μg/ml) or (**B**) flagellin (10 μg/ml) for 6 hours. The cell culture supernatants and cell lysates were analyzed for IL-1β, pro-IL-1β, cleaved caspase-1, and pro-caspase-1 by immunoblot assay.
